# Epidemiological Study of Tricuspid Regurgitation After Cardiac Transplantation. Does it Influence Survival?

**DOI:** 10.3389/ti.2022.10197

**Published:** 2022-03-21

**Authors:** Raquel López-Vilella, María J. Paniagua-Martín, Francisco González-Vílchez, Víctor Donoso Trenado, Eduardo Barge-Caballero, Ignacio Sánchez-Lázaro, Ana V. Aller Fernández, Luis Martínez-Dolz, María G. Crespo-Leiro, Luis Almenar-Bonet

**Affiliations:** ^1^ Heart Failure and Transplantation Unit, Hospital Universitario y Politécnico La Fe, Valencia, Spain; ^2^ Department of Cardiology, Hospital Universitario y Politécnico La Fe, Valencia, Spain; ^3^ Department of Cardiology, Complejo Hospitalario Universitario de A Coruña, Servicio Galego de Saúde (SERGAS), A Coruña, Spain; ^4^ Hospital Universitario Marqués de Valdecilla, Santander, Spain; ^5^ Centro de Investigación Biomédica en Red de Enfermedades Cardiovasculares (CIBERCV), Instituto de Salud Carlos III, Madrid, Spain; ^6^ Department of Intensive Medicine, Complejo Hospitalario Universitario de A Coruña, Servicio Galego de Saúde (SERGAS), A Coruña, Spain; ^7^ Universidade da Coruña (UDC), A Coruña, Spain; ^8^ Department of Medicine, Universidad de Valencia, Valencia, Spain

**Keywords:** heart transplantation, survival, prognosis, tricuspid regurgitation, aetiology

## Abstract

**Background:** Tricuspid valve disease is the most frequent valvulopathy after heart transplantation (HTx). Evidence for the negative effect of post-transplant tricuspid regurgitation (TR) on survival is contradictory. The aim of this study was to analyze the causes of post-transplant TR and its effect on overall mortality.

**Methods:** This is a retrospective observational study of all transplants performed in two Spanish centers (1009 patients) between 2000 and 2019. Of the total number of patients, 809 had no TR or mild TR and 200 had moderate or severe TR. The etiology of TR was analyzed in all cases.

**Results:** The prevalence of moderate and severe TR was 19.8%. The risk of mortality was greater when TR was caused by early primary graft failure (PGF) or rejection (*p* < 0.05). TR incidence was related to etiology: incidence of PGF-induced TR was higher in the first period, while TR due to rejection and undefined causes occurred more frequently in three periods: in the first year, in the 10–14-year period following HTx, and in the long term (16–18 years). In the multivariable analysis, TR was significantly associated with mortality/retransplantation (HR:1.04, 95% CI:1.01–1.07, p:0.02).

**Conclusion:** The development of TR after HTx is relatively frequent. The annual incidence depends on TR severity and etiology. The risk of mortality is greater in severe TR due to PGF or rejection.

## Graphical Abstract

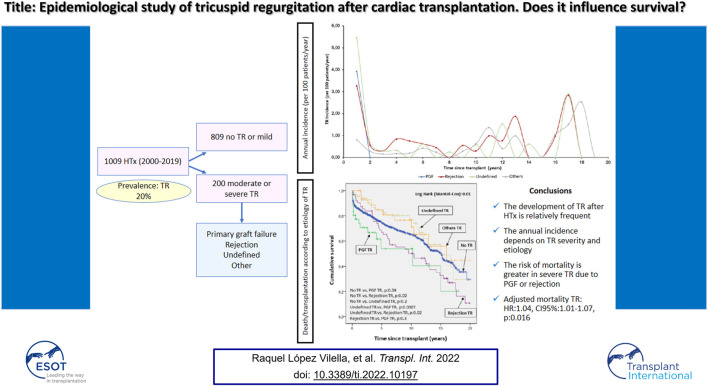

## Introduction

Heart transplantation (HTx) remains the treatment of choice for end-stage heart failure (HF) (1). Overall, outcomes of HTx have improved in recent decades (2); however, a series of short, medium, and long-term complications continue to have an impact on prognosis. Tricuspid regurgitation (TR) is the most frequent valve disease after orthotopic HTx in both the short and long-term, and has a prevalence ranging from 19% to 84%, depending on the series (3). In most cases, TR is mild and asymptomatic, but some cases of moderate or severe TR are associated with increased morbidity and mortality (3–7). However, the prognostic implications of TR after heart transplantation is not clearly defined. Some authors associate post-transplant TR with adverse outcomes, while according to others most cases of significant TR resolve within 1 year of transplant (8, 9). Identifying patients with significant TR who will develop such complications remains challenging, and warrants further clinical investigation. It has been suggested that the development and prognostic impact of TR depends not only on its severity, but also on its etiology. Thus, there is a type of early post-operative TR caused by primary graft failure (PGF) with or without pulmonary hypertension (7, 10, 11), and another later type of TR associated with rejection or other causes (9). In any event, TR, its causes, and its prognostic implications have not hitherto been studied in detail.

We hypothesized that not all causes of TR have the same effect on mortality or the same evolution in transplant patients. Studying the evolution of TR after heart transplantation and both its cause-specific and general impact would improve the characterization of this valvular disease, and help identify the therapeutic approach and follow-up that would be most beneficial in these patients.

We performed an epidemiological study in a large series of heart transplant patients to determine the prevalence of TR and its influence on long-term mortality. The secondary objective was to perform a subanalysis of the most common etiologies of TR, its differential characteristics, and its etiology-specific impact on survival after transplantation.

### Patients and Methods

**FIGURE 1:**
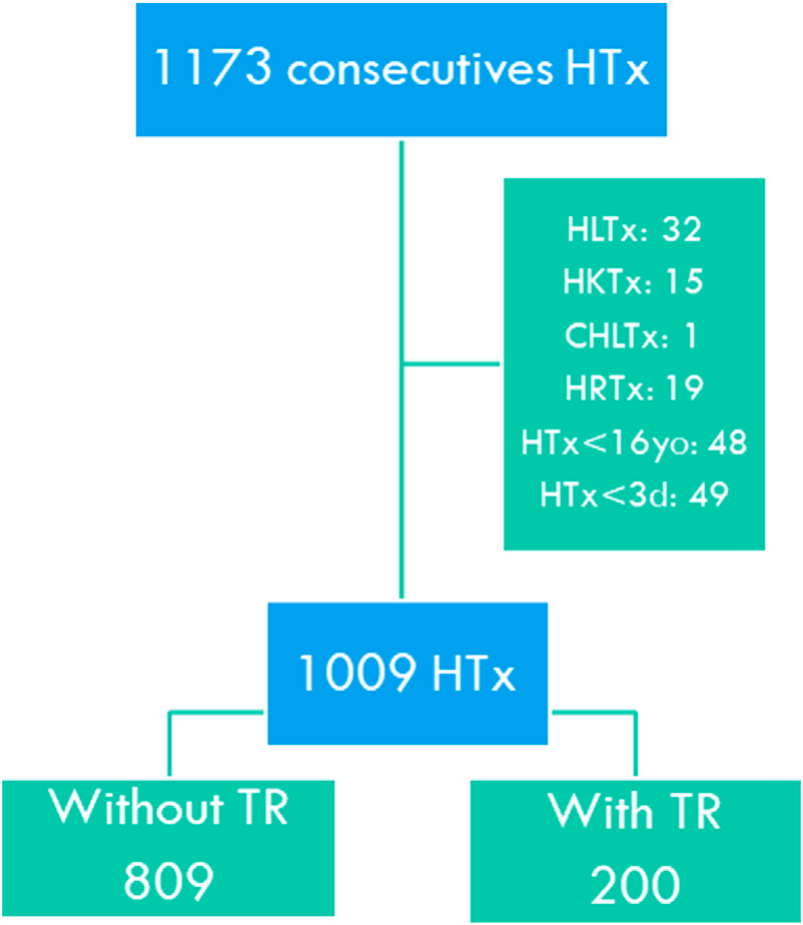
Study flow chart. HTx, Heart transplant; HLTx, Combined heart-lung transplant; HKTx, Combined heart-kidney transplant; CHLTx, Combined heart-liver transplant; HRTx, Heart retransplant; HTx <16yo, Pediatric heart transplant; HTx <3d, Death within the first 3 days of transplantation. TR, Tricuspid regurgitation.

We performed a retrospective observational study that included all patients who had undergone HTx in two Spanish centers between 1 January, 2000 and 31 December, 2019. Multi-organ transplants, retransplants, patients under 16 years at the time of transplantation, and patients who died during the first 72 h of transplantation were excluded (Figure 1).

TR was grouped according to etiology: PGF, acute rejection, undefined causes, and others. TR due to undefined cause was defined as functional TR with no identifiable cause. All variables included in the Spanish Registry of Cardiac Transplants, defined elsewhere, were evaluated (12). The donor-recipient body size match was analyzed using the predicted right, left, and total ventricular mass, which has proven to be the body metric with the greatest prognostic value (13). Glomerular filtration rate was estimated using the formula recommended by the Chronic Kidney Disease Epidemiology Collaboration (14). The presence of TR, time of appearance after transplantation, severity of ventricular dysfunction, evolution of valve disease, clinical course, treatment, and patient status at the end of follow-up were also analyzed.

Information on TR grade was obtained from echocardiography reports following the recommendations in force in each time period. Four grades (absent, mild, moderate, or severe) were analyzed. The TR group comprised exclusively moderate and severe grade regurgitations. The cardiologists used semi-quantitative or qualitative parameters to evaluate tricuspid regurgitation, depending on the protocol in place in each center and the clinical status of the patient. Reduced ventricular function and chamber dilation were also diagnosed (1). Right ventricular dysfunction and right ventricular dilatation were evaluated both qualitatively and quantitatively by measurement of the basal diameter of the cavity, S wave, and systolic excursion of the tricuspid annulus (TAPSE). Echocardiography is a technique that is used systematically at any time during the follow-up of the cardiac transplant patient. In this series, the echocardiography protocol consisted of a transthoracic echocardiogram performed almost daily in the early phase of the transplant, during scheduled biopsies (7–9 during the first year), and during follow-up (every 4–6 months), according to the protocol implemented in each center. Echocardiography was also performed whenever instability, clinical deterioration, or valve involvement was suspected. It is important to emphasize that clinical guidelines on the measurement and quantification of tricuspid regurgitation did not change significantly over the course of the study. Both study centers followed the same measurement guidelines.

The study was approved by the Biomedical Research Ethics Committee of both participating centers, and the ethical principles for medical research in human subjects defined by the Declaration of Helsinki were followed.

### Statistical Analysis

Continuous variables are summarized as median (interquartile range [IQR]), as all of them showed a non-normal distribution (Kolmogorov-Smirnov test). Categorical variables are summarized as frequency (percentage). Group variables were compared using the Mann-Whitney test in the case of continuous variables and using Fisher’s exact test or the Chi-square test in the case of categorical variables.

A multivariate Cox proportional hazard regression model was used to determine independent predictors of TR, introducing as predictors the variables that showed a *p* value <0.10 in the univariable analysis.

The main outcome was a composite of all-cause mortality or cardiac retransplant. The association between the occurrence of TR and mortality/transplantation was first analyzed by the Kaplan-Meier procedure and the differences between groups by the Log-Rank test. As both graphs and the Schoenfeld residual test showed that TR violated the proportional hazard assumption, it was considered a time-dependent variable for the purpose of univariate and multivariate analysis. Association with the outcome was analyzed by means of Cox proportional hazards regression. In the multivariate analysis, variables that showed a significance level *p* < 0.10 in the univariable analysis were introduced as independent variables, including TR as a time-dependent variable.

All tests were two-tailed, establishing statistical significance for a *p* value <0.05. The analyses were performed with IBM SPSS Statistics Version 27 ^®^.

## Results

A total of 200 (19.8%) of the 1009 patients included developed moderate or severe TR during follow-up. TR was graded as moderate in 133 recipients (13.2%) and severe in 67 (6.6%).

### Baseline Characteristics

Pre-transplant clinical characteristics for the entire population and by TR group are summarized in Table 1. Patients who developed TR were younger, had higher pre-transplant bilirubin, and were transplanted from older and female donors more frequently. Donor-recipient sex mismatch was more frequent (higher percentage of female donor to male recipient grafts), and the donor-recipient-predicted right ventricular mass ratio was lower in patients developing TR.

**TABLE 1:** Baseline characteristics.

	No TR (*n*: 809)	TR (*n*: 200)	*p* value	Total population (*n*: 1009)
Recipient				
Age (years)	56.0 (48.5–63.0)	55.0 (45.0–61.0)	0.017	56.0 (48.0–62.0)
Female sex, n (%)	143 (17.7)	42 (21.1)	0.22	185 (18.4)
Etiology, n (%)			0.33	
Ischemic	335 (41.4)	72 (36.0)		407 (40.3)
Dilated	329 (40.7)	86 (43.0)		415 (41.1)
Other	145 (17.9)	42 (21.0)		187 (18.5)
Body mass index (Kg/m^2^)	25.4 (23.1–28.4)	24.8 (22.5–27.8)	0.058	25.2 (23.0–28.3)
Creatinine (mg/dl)	1.1 (0.9–1.4)	1.2 (0.9–1.4)	0.31	1.13 (0.9–1.4)
Glomerular filtration rate (mL/min/1.73 m^2^)	69.6 (51.7–90.6)	67.8 (49.6–90.7)	0.45	69.5 (51.5–90.6)
Bilirubin (mg/dl)	1.0 (0.6–1.6)	1.2 (0.7–1.9)	0.024	1.0 (0.6–1.7)
PVR (Wood U.)	2.1 (1.3–3.0)	2.2 (1.5–3.1)	0.19	2.1 (1.3–3.0)
Pretransplant infection, n (%)	70 (8.7)	15 (7.5)	0.67	85 (8.4)
Diabetes Mellitus, n (%)	111 (13.7)	35 (17.5)	0.18	146 (14.5)
COPD, n (%)	83 (12.2)	23 (13.2)	0.70	106 (12.4)
Positive CMV serology, n (%)	643 (81.4)	167 (85.2)	0.25	810 (82.2)
Peripheral vascular disease, n (%)	37 (4.6)	10 (5.0)	0.85	47 (4.7)
Mechanical ventilation, n (%)	122 (15.1)	24 (12.1)	0.31	146 (14.5)
Circulatory support, n (%)			0.29	
No	603 (74.9)	150 (75.0)		753 (74.9)
IABP	88 (10.9)	26 (13.0)		114 (11.3)
ECMO	69 (8.6)	10 (5.0)		79 (7.9)
VAD	45 (5.6)	14 (7.0)		59 (5.9)
Previous sternotomy	144 (17.8)	41 (20.5)	0.36	185 (18.4)
Pretransplant neoplasy, n (%)	27 (3.4)	4 (2.1)	0.49	31 (3.1)
Donor				
Age (years)	44.0 (31.0–51.0)	47.0 (38.0–55.0)	<0.001	44 (32–52)
Female sex, n (%)	238 (29.5)	95 (47.5)	<0.001	333 (33.0)
Body mass index (Kg/m^2^)	25.4 (23.9–27.7)	25.6 (23.9–27.8)	0.71	25.4 (23.9–27.7)
Positive CMV serology, n (%)	592 (76.4)	155 (81.2)	0.18	747 (77.3)
Predonation cardiac arrest, n (%)	56 (7.1)	18 (9.2)	0.36	74 (7.5)
Cause of death, n (%)			0.059	
Trauma	273 (33.7)	50 (25.0)		323 (32.0)
Cerebrovascular accident	364 (45.0)	103 (51.5)		467 (46.3)
Other	172 (21.3)	47 (23.5)		219 (21.7)
Donor-recipient interaction				
Sex mismatch, n (%)			<0.001	
No mismatch	573 (70.9)	112 (56.0)		685 (68.0)
Donor male/Recipient female	70 (8.7)	18 (9.0)		88 (8.7)
Donor female/Recipient male	165 (20.4)	70 (35.0)		235 (23.3)
CMV serology mismatch, n (%)			0.41	
No mismatch	506 (66.8)	135 (71.8)		641 (67.8)
Donor (-)/Recipient (+)	145 (19.1)	30 (16.0)		175 (18.5)
Donor (+)/Recicipient (-)	107 (14.1)	23 (12.2)		130 (13.7)
Donor-recipient PRVM ratio	1.12 (1.00–1.27)	1.06 (0.94–1.17)	<0.001	1.11 (0.99–1.25)
Donor-recipient PHM ratio	1.0 (1.0–1.1)	1.0 (1.0–1.1)	0.27	1.0 (1.0–1.2)
Surgical procedure				
Urgent code, n (%)^a^	264 (32.6)	68 (34.0)	0.74	332 (32.9)
Cold ischemia duration (min)	180 (115–222)	194 (114–248)	0.08	180.0 (115–227)
Bicaval technique, n (%)	660 (88.8)	161 (85.2)	0.17	821 (88.1)
Follow up				
Time (years)	5.8 (1.8–12.0)	6.3 (2.4–11.8)	0.27	5.9 (1.9–11.9)
Status, n (%)			0.15	
Alive	497 (61.4)	109 (54.5)		606 (60.1)
Dead	305 (37.3)	88 (44.0)		393 (38.9)
Retransplanted	7 (0.9)	3 (1.5)		10 (1.0)

a Urgent Code transplantation was performed in severe cardiogenic shock.

CMV, cytomegalovirus; COPD, chronic obstructive pulmonary Disease; ECMO, extracorporeal membrane oxygenation; IABP, Intra-Aortic Balloon Pump; PHM, predicted heart mass; PRVM, predicted right ventricular mass; PVR, pulmonary vascular resistance; TR, tricuspid regurgitation; VAD, ventricular assist device.

### Etiological Characteristics of Tricuspid Regurgitation

Differences in TR characteristics according to etiology are summarized in Table 2. The most frequent etiology was undefined causes, followed by acute rejection. PGF-induced TR resulted more frequently in dilation and dysfunction of the right ventricle. All types of TR improved over time and subsided on echocardiography. In the group of other etiologies, TR was more frequently associated with pulmonary hypertension, coronary allograft vasculopathy, pacemaker implantation, and biopsy complications (Table 3).

**TABLE 2:** Characteristics of tricuspid regurgitation in transplanted patients according to the etiological types.

	Primary graft failure (*n*: 35)	Acute rejection (*n*: 64)	Undefined (*n*: 72)	Other (*n*: 29)		*p* value
Chronology	Very early	Late and very late	Very late	Very late		
Time of appearance	First year	1–18 years	11–18 years	10–18 years		
Prevalence, n (%)	35 (17.5)	64 (32.0)	72 (36.0)	29 (14.5)		0.008
Grading of TR						0.01
Moderate	19 (54.3)	40 (62.5)	58 (80.6)	16 (55.2)		
Severe	16 (45.7)	24 (37.5)	14 (19.4)	13 (44.8)		
Right ventricular dilatation	20 (57.1)	15 (23.4)	11 (15.3)	13 (41.8)		<0.001
Right ventricular dysfunction	31 (88.6)	32 (50.0)	9 (12.5)	14 (48.3)		<0.001
Left ventricular dysfunction	8 (22.9)	21 (32.8)	1 (1.4)	4 (13.8)		<0.001
Echocardiography time course						0.01
Improvement	29 (82.9)	46 (71.9)	58 (82.9)	16 (55.2)		
Stable	6 (17.1)	12 (18.8)	12 (17.1)	8 (27.6)		
Deterioration	0 (0.0)	6 (9.5)	0 (0.0)	5 (16.2)		
Congestive signs	14 (40.0)	42 (65.6)	22 (30.6)	12 (41.4)		0.001
Clinical course of congestive signs^a^						0.005
Improvement	9 (64.3)	23 (54.8)	15 (68.2)	5 (41.7)		
Stable	1 (5.0)	14 (33.3)	6 (27.3)	1 (8.3)		
Deterioration	0 (0.0)	5 (11.9)	1 (4.5)	6 (50.0)		
Number of diuretics^b^						<0.001
0	17 (48.6)	23 (35.9)	47 (65.3)	13 (44.8)		
1	18 (51.4)	29 (45.3)	21 (29.2)	10 (34.5)		
2	0 (0.0)	11 (17.2)	4 (5.6)	3 (10.3)		
3	0 (0.0)	1 (1.6)	0 (0.0)	3 (10.3)		
Treatment						<0.001
No/symptomatic	0 (0.0)	2 (3.1)	71 (98.6)	16 (55.2)		
Etiological	35 (100.0)	61 (95.3)	0 (0.0)	11 (37.9)		
Retransplantation	0 (0.0)	1 (1.6)	0 (0.0)	1 (3.4)		
Coronary stent	0 (0.0)	0 (0.0)	0 (0.0)	1 (3.4)		
Annuloplasty	0 (0.0)	0 (0.0)	1 (1.4)	0 (0.0)		

a Right-sided congestive signs that can be attributed to tricuspid regurgitation have been analyzed.

b Including any type of diuretic that each patient was prescribed (loop diuretics, thiazides, acetazolamide and/or tolvaptan).

TR, tricuspid regurgitation.

**TABLE 3:** Causes of post-transplant tricuspid regurgitation in the group “Other".

	N	%
Pulmonary hypertension	9	31.0
Cardiac allograft vasculopathy	7	24.1
Pacemaker Electrode	4	13.8
Biopsy complication	3	10.3
Chronic renal insufficiency	2	6.9
Severe pericardial effusion^a^	1	3.4
Valve prolapse	1	3.4
Atrial tachycardia	1	3.4
Massive Pulmonary Embolism	1	3.4

a Severe pericardial effusion with distortion of the geometry of the right ventricular cavity and the valve annulus.

### Incidence of Tricuspid Regurgitation

**FIGURE 2:**
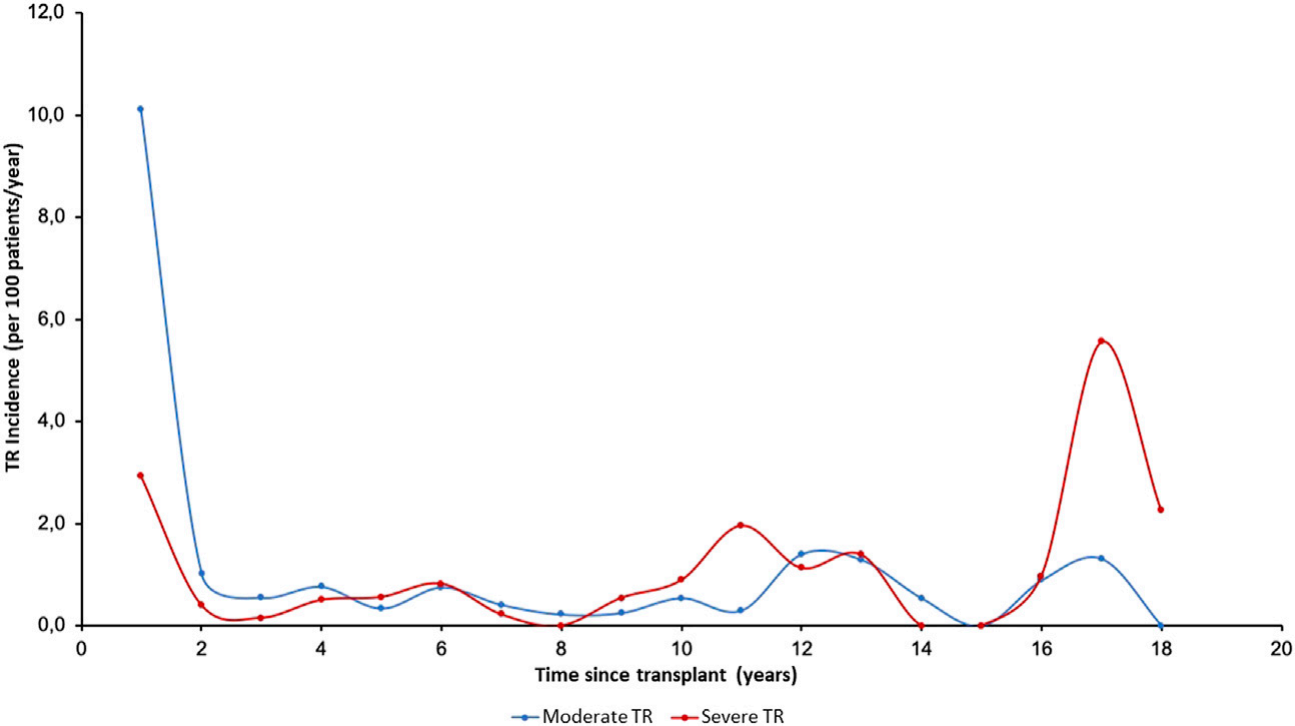
Annual incidence (per 100 patients/year) of tricuspid valve disease in follow-up according to severity. TR, Tricuspid regurgitation.

The incidence of TR (per 100 patient-years) over the 20 years of post-transplant follow-up according to the degree of regurgitation is shown in Figure 2. Median time to overall TR was 0.57 years (IQR, 0.06–5.60 years); this was significantly lower in moderate TR (median: 0.12 years [0.04–1.78 years]) compared to severe TR (median: 5.24 years [1.30–10.90 years]; *p* < 0.001).

**FIGURE 3:**
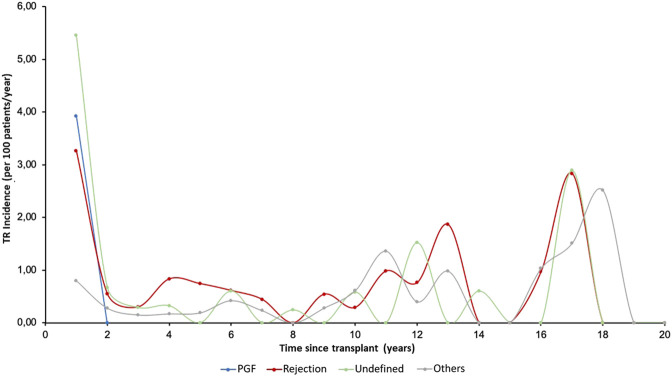
Annual incidence (per 100 patients/year) of tricuspid valve disease in follow-up according to etiology. PGF, Primary graft failure; TR, Tricuspid regurgitation.

The incidence of moderate TR was highest in the first period after HTx, while severe TR generally appeared later. Figure 3 shows the temporal distribution of the appearance of TR according to etiology. The incidence of PGF-induced TR was highest in the first period while TR due to rejection and undefined causes occurred more frequently in three periods: in the first year, in the 10–14-year period after HTx, and in the long term (16–18 years), showing a triphasic distribution.

### Independent Predictors of Post-Transplant Tricuspid Regurgitation

**FIGURE 4:**
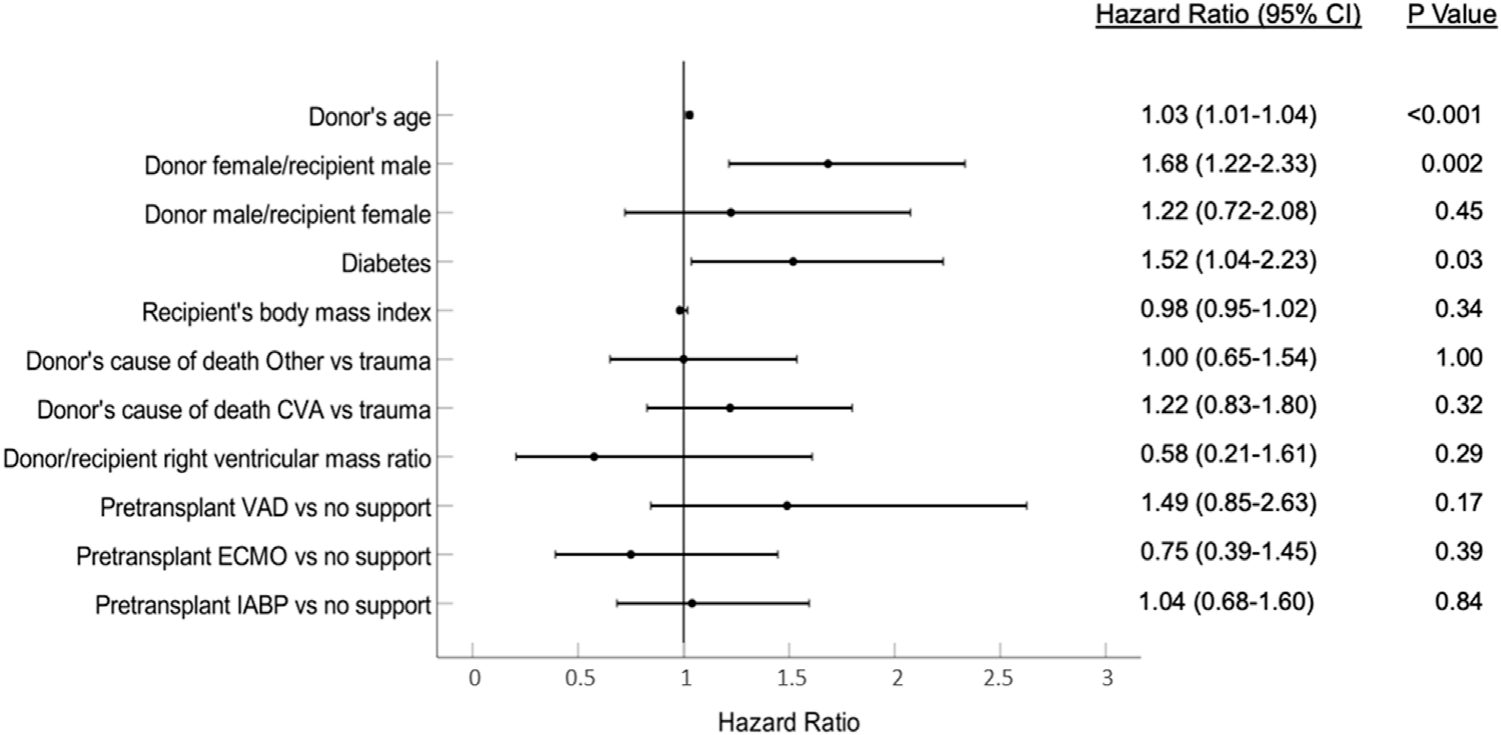
Variables associated with the development of post-transplantation tricuspid regurgitation. CVA, Cerebrovascular accident; ECMO, Extracorporeal membrane oxygenation; IABP, Intra-aortic balloon pump; VAD, Ventricular assist device.

Univariate associations with the development of moderate-severe TR are summarized in Supplementary Table S1. Diabetes, ventricular assist device prior to heart transplantation, higher donor age, female donors, donor cause of death other than trauma, and donor-recipient sex mismatch (female donor for male recipient) were risk factors. Higher recipient body mass index and higher donor-recipient-predicted right ventricular mass ratio were protective factors. In the multivariate analysis, only diabetes, donor age, and donor-recipient sex mismatch (female donor for male recipient) were independently associated with development of TR (Figure 4).

### Survival

**FIGURE 5:**
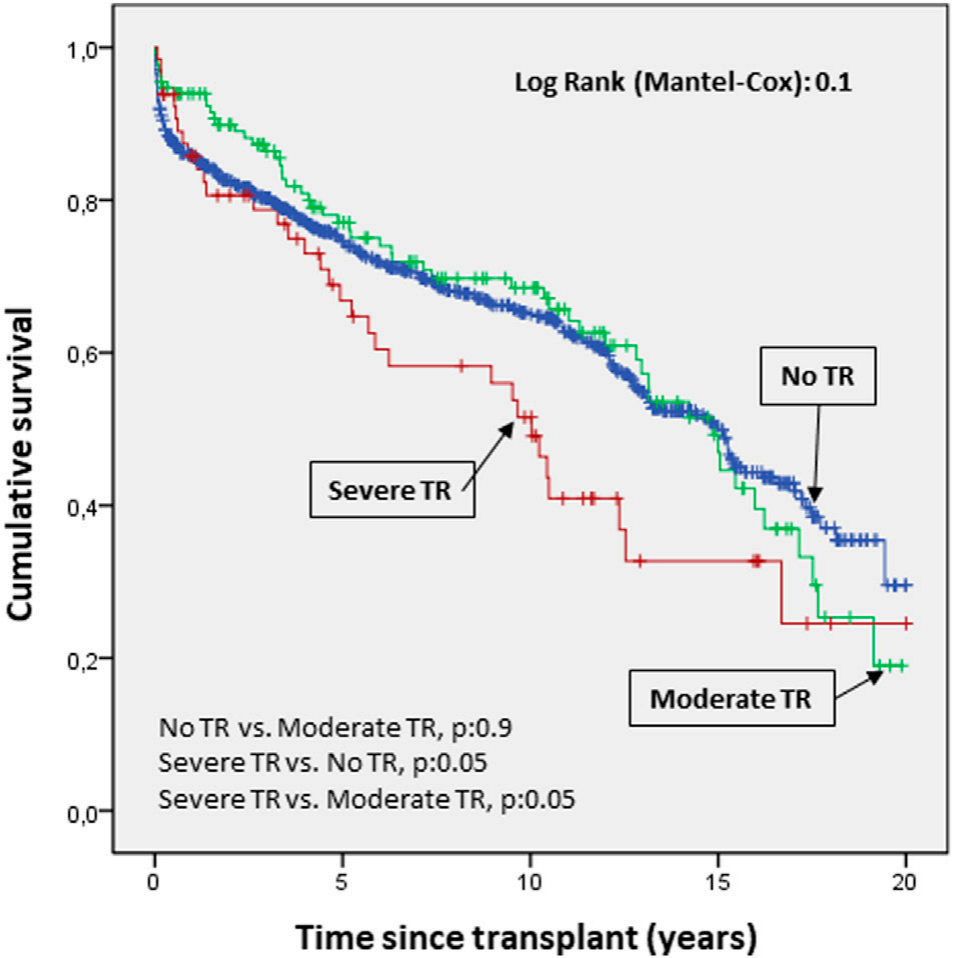
Cumulative probability of mortality/transplantation according to severity of tricuspid valve disease. The probability of survival according to the severity of regurgitation showed a clear trend toward higher mortality in severe versus moderate tricuspid regurgitation and no regurgitation.

There were 393 deaths and 10 retransplants during a median follow-up of 5.9 years (IQR, 1.9–11.9). Survival analysis according to TR severity showed a higher rate of mortality (p:0.05) for severe TR compared to moderate TR and no TR (Figure 5).

**FIGURE 6:**
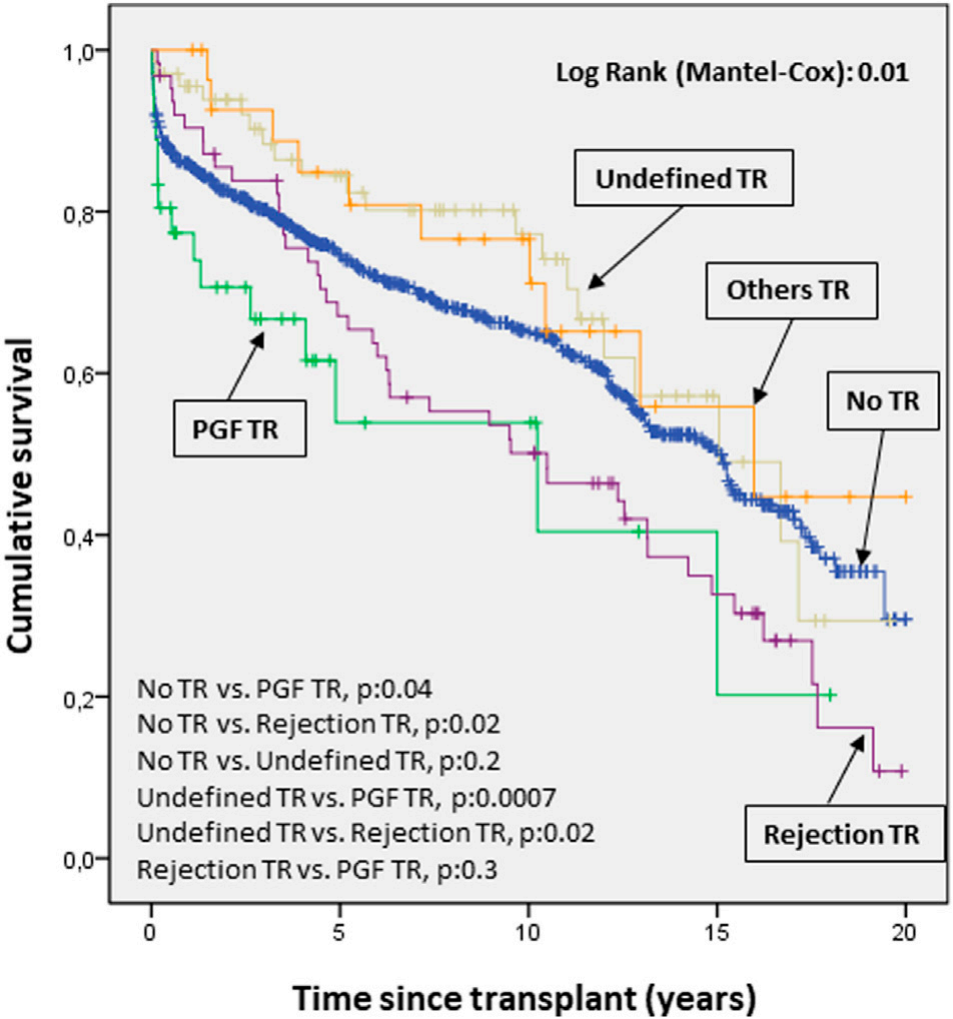
Cumulative probability of death/transplantation according to etiology of tricuspid valve disease. The survival curves for mortality/transplantation show a significantly worse prognosis (*p* < 0.05) when tricuspid disease was due to primary graft failure and rejection compared to other causes. PGF, Primary graft failure; TR, Tricuspid regurgitation.

The survival curves for mortality/transplantation showed a significantly worse prognosis when TR was due to PGF and rejection compared to other causes (p 0.04 and 0.02, respectively, Figure 6).

### Prognostic Impact of Post-transplant Tricuspid Regurgitation

**FIGURE 7:**
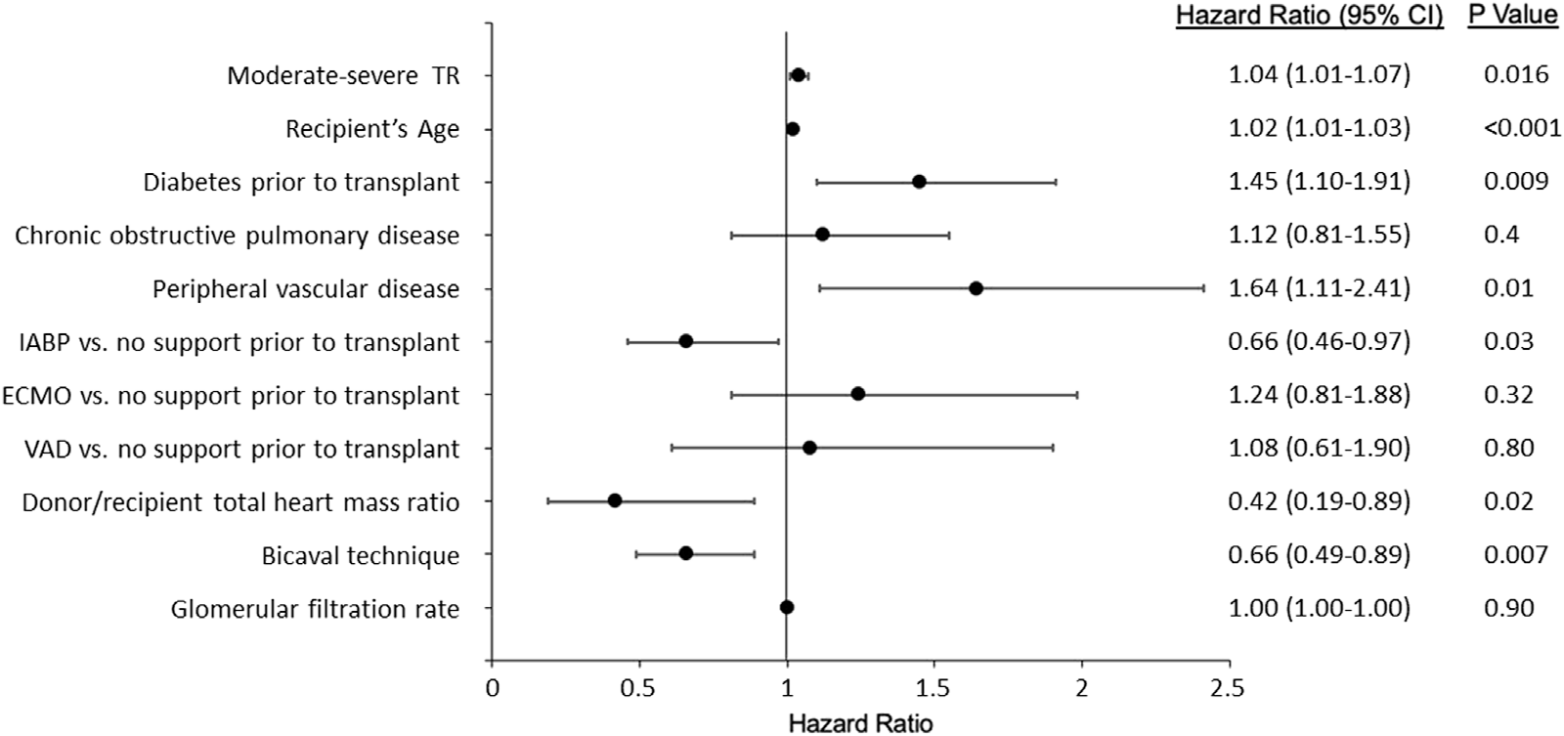
Variables associated with mortality/transplantation. IABP, Intra-aortic balloon pump; ECMO, Extracorporeal membrane oxygenation; VAD, Ventricular assist device; TR, Tricuspid regurgitation.

In the univariate analysis (Supplementary Table S2), post-transplantation TR was associated with higher mortality/retransplantation (HR: 1.04; 95% CI: 1.01–1.07; p: 0.02). The variables significantly associated with a higher risk for mortality/transplantation in the multivariable analysis were the presence of moderate to severe TR, recipient age at transplant, pre-transplant diabetes, and peripheral vascular disease. Protective factors were bicaval technique (versus biatrial technique), use of intra-aortic balloon pump (versus no pump), and a higher donor-recipient heart mass ratio. These results are shown in Figure 7.

## Discussion

TR is the most prevalent valve disease after HTx3. Its causes vary and each may have different prognostic implications. Some studies have analyzed the prevalence this valve disease and its implication in survival. However, the incidence, time of appearance, and TR etiology-specific prognostic implications have never been fully defined. In this study, we sought to clarify these questions by analyzing a large series of heart transplant patients from two Spanish centers with high transplant activity. We found that the prevalence of post-transplant moderate or severe TR was nearly 20%, and that the most frequent cause was functional, i.e., no organic valve alteration and no specific cause for TR. TR appearing in the early stages of PGF and during acute rejection had the highest risk for mortality. TR was also found to be an independent predictor of mortality, and its appearance was related to donor age and donor-recipient sex mismatch (specifically, female donor for male recipient).

A total of 1,009 cardiac transplants were included in this study, constituting the largest cohort so far. A total of 200 patients in our series presented at least moderate TR during their evolution–a prevalence of 19.8%, similar to that found in previous publications (15). In this study, the pre-transplant clinical characteristics of patients in both groups were basically similar. Patients who developed TR were younger and had higher bilirubin; however, these differences were not clinically relevant. In addition, patients who developed TR had a lower donor-recipient-predicted right ventricular mass ratio, as observed in the univariate analysis. This index has predictive value for mortality after heart transplantation, but it has not hitherto been associated with the appearance of TR (13). Nevertheless, our multivariate analysis showed that the development of TR was independently associated with diabetes, donor age, and donor-recipient sex mismatch (female donor for male recipient). Previous studies have described a correlation between the appearance of TR and donor heart size - recipient pericardial cavity mismatch (16, 17), and female recipient has been shown to be an independent predictor of rapid progression of TR (18). However, the correlation between TR and sex mismatch, and between diabetes in the recipient and post-transplant TR have not hitherto been described in the literature. These findings could help optimize donor/recipient selection and reduce the risk of post-transplantation TR.

The etiology of TR after HTx has not been completely clarified. This is the first study to address both this issue and the timing time of TR onset according to etiology. Thus, the undefined etiology was the most frequent (functional TR with no identifiable cause) followed by acute rejection. PGR-induced TR showed the closest correlation with right ventricle dilation and presence of biventricular dysfunction, followed by rejection-induced TR, which was also associated with ventricular dysfunction. The timing of TR onset is also related to its etiology. Our findings show that TR associated with early primary graft failure is the first to appear, while TR due to rejection and undefined cause is triphasic, with an initial incidence (first year), another incidence in the medium term (10–14 years post-HTx) and finally, a long-term incidence (16–18 years). Few studies have analyzed the predictors of early vs late TR. Williams et al. reported a significant increase in TR on echocardiography performed at week 1 compared with the same study performed at 2.4 ± 1.3 years after HTx, with incidence rates of 63% and 71%, respectively (19). In another study, the incidence of severe TR increased from 5% at 1 year up to 50% 4 years after transplantation (17, 20). A previous study reported that the development of early TR was correlated with allograft rejection, high transpulmonary gradient, and high pulmonary vascular resistance, while the risk factors for late TR were biatrial surgical technique, the number of rejections, and the total number of endomyocardial biopsies performed (21). All these findings confirm that TR is a complication that can appear either very early after HTx or many years after the intervention. In fact, it appears to be a dynamic condition; severe early TR has been shown to subside 1 year after transplantation in more than 91% of recipients (22). For this reason, the reported incidence of TR is higher in the first post-transplant year, although there continues to be a risk of developing TR thereafter. This late risk can be aggravated by repeated endomyocardial biopsies (6, 21). In our study, echocardiographic study of most cases of TR showed improvement over time.

Regarding the impact of post-transplantation TR on mortality, the mortality/transplantation survival curves showed a clear trend towards higher mortality in severe TR compared with moderate TR and no TR. In previous studies, TR has been associated with decreased long-term survival after heart transplantation. However, although these data are contradictory (8, 9, 22, 23), in general, most authors agree that this valve disease is predictive of mortality (6). In some studies, even intraoperative TR was associated with increased mortality in HTx patients (8). In this study, the variables significantly associated with an increased risk for mortality/transplantation in the multivariable analysis were presence of moderate-severe TR, recipient age, pre-transplant diabetes, and peripheral vascular disease. Protective factors were bicaval technique (versus biatrial technique), use of an intraaortic balloon pump (versus no pump), and a higher donor/recipient-predicted heart mass ratio. Previous studies have reported that the likelihood of developing TR was greater if HTx is performed using the biatrial technique compared to the bicaval technique. This may be due to the fact that the traditional technique (biatrial) significantly alters atrial geometry, resulting in deterioration of valve integrity (24–26). Regarding the finding of diabetes mellitus as a risk factor for the development of post-transplant TR, this is a finding that has not been described in the literature. One possible explanation could be that the vascular and microvascular involvement of these patients has an impact on ventricular morphology. However, this is only a hypothesis; it is possible that this is a clinically irrelevant finding, as it is not associated with the other independent predictors of the development of TR, which mainly refer to the donor.

Finally, the survival curves for mortality/transplantation showed a significantly worse prognosis when the etiology of TR was due to PGF and rejection compared to other causes. TR patients have similar long-term prognosis compared to patients without TR.

These data are consistent with the known prognosis for both conditions. Currently, PGF is one of the most frequent causes of mortality, especially in the first month after transplantation, while rejection is the second most frequent cause of death between the first and fifth year after transplantation (2).

This study has some limitations, especially due to its retrospective nature. The protocols for performing the echocardiographic study varied slightly, as they were performed in two different centers. Moreover, patients who died within the first 3 days of transplantation had to be ruled out because in these cases echocardiographic studies, especially in the presence of severe PGF, were focused primarily on assessing the degree of ventricular dysfunction, not the presence of tricuspid valve disease, and there were no data on TR in these echocardiography reports. Nevertheless, the major strength of the study is the large sample size and the detailed description of causes, time-related characteristics, and the prognostic impact of TR. The size of our series - 200 cases of tricuspid valve disease collected over 20 years of transplant activity in two centers with a high number of annual implants–supports the reliability of our findings. Furthermore, we have not found any previous studies with such a detailed description of the incidence of valve disease, its prognostic importance, and its influence on mortality. For all these reasons, we believe our conclusions can safely be extrapolated to other settings.

Based on our findings, we can conclude that the prevalence of moderate and severe tricuspid regurgitation is close to 20%, with a variable annual incidence depending on the severity and etiology of the valve disease. This valvulopathy, especially in its severe manifestation, is associated with a high risk of mortality, particularly when it is due to rejection and primary graft failure. The multivariate analysis shows a significant association between mortality/transplantation and TR.

## Data Availability

The raw data supporting the conclusion of this article will be made available by the authors, without undue reservation.

## Abbreviations

HF, heart failure; IQR, interquartile range; HTx, heart transplantation; PGF, primary graft failure; TR, tricuspid regurgitation.
