# Supplementary material for: Epidemiological Study of Tricuspid Regurgitation After Cardiac Transplantation. Does it Influence Survival?
Source: Transpl Int. 2022 Mar 21;35:10197. doi: 10.3389/ti.2022.10197 (PMC8979064; doi:10.3389/ti.2022.10197)
Supplement: Supplementary file 1 [file DataSheet1.PDF]

Supplementary material. Table S1. Univariable analysis for the development of post-transplant tricuspid regurgitation.

	HR	95% CI	P Value
**Recipient characteristics**			
Age (years)	0.99	0.98-1.01	0.41
Female sex	1.21	0.86-1.70	0.28
Etiology			
Ischemic	1		
Dilated	1.18	0.86-1,61	0.31
Other	1.28	0.87-1.88	0.21
Body mass index (Kg/m^2)	0.97	0.94-1.00	0.08
Creatinine	1.21	0.92-1.59	0.21
Glomerular filtration rate (mL/min/1.73 m^2)	1.00	0.99-1.00	0.26
Bilirubin (mg/dL)			
Pulmonary vascular resistance (Wood U.)	1.03	0.94-1.13	0.58
Infection	0.92	0.54-1.56	0.75
Diabetes	1.66	1.15-2.40	0.007
COPD	1.21	0.78-1.89	0.39
Positive CMV serology	1.22	0.82-1.80	0.33
Peripheral vascular disease	1.35	0.72-2.56	0.35

Mechanical ventilation	0.82	0.54-1.26	0.37
Pretransplant circulatory support			
No	1		
IABP	1.16	0.77-1.77	0.48
ECMO	0.83	0.44-1.58	0.57
VAD	2.01	1.15-3.50	0.01
Previous sternotomy	1.12	0.79-1.58	0.52
Pretransplant neoplasy	0.79	0.29-2.14	0.65
**Donor characteristics**			
Age (years)	1.04	1.03-1.05	<0.001
Female sex	2.02	1.53-2.67	<0.001
Body mass index (Kg/m^2)	1.02	0.98-1.06	0.28
Positive CMV serology	1.18	0.83-1.69	0.36
Predonation cardiac arrest	1.32	0.81-2.14	0.27
Cause of death			
Trauma	1		
Cerebrovascular accident	2.11	1.49-2.99	<0.001
Other	1.60	1.07-2.39	0.02
**Donor-recipient interaction**			
Sex mismatch			
No	1		
Donor male/recipient female	1.18	0.71-1.96	0.53

Donor female/recipient male	1.99	1.49-2.66	<0.001
CMV serology mismatch			
No	1		
Donor negative/recipient positive	0.83	0.56-1.22	0.34
Donor positive/recipient negative	0.88	0.55-1.40	0.58
Predicted right ventricular mass ratio	0.15	0.07-0.32	<0.001
Predicted total heart mass ratio	0.52	0.18-1.51	0.23
Surgical procedure			
Urgent code	1.09	0.81-1.47	0.57
Cold ischemia duration (min)	1.00	1.00-1.00	0.24
Bicaval surgical technique	0.77	0.51-1.17	0.22
CMV: Cytomegalovirus; COPD: Chronic Obstructive Pulmonary Disease; ECMO: Extracorporeal Membrane Oxygenation; IABP: Intra-Aortic Balloon Pump; VAD: Ventricular Assist Device.			

Supplementary material. Table S2. Univariable analysis for mortality.

	HR	95% CI	P Value
**Recipient characteristics**			
Age (years)	1.02	1.01-1.03	<0.001
Female sex	0.81	0.62-1.06	0.13
Etiology			
Ischemic	1		
Dilated	0.90	0.73-1.12	0.35
Other	0.98	0.75-1.29	0.90
Body mass index (Kg/m^2)	1.01	0.99-1.04	0.34
Creatinine	1.14	0.94-1.40	0.19
Glomerular filtration rate (mL/min/1.73 m^2)	1.00	0.99-1.00	0.06
Bilirubin (mg/dL)			
Pulmonary vascular resistance (Wood U.)	0.96	0.89-1.04	0.34
Infection	0.87	0.59-1.27	0.46
Diabetes	1.67	1.27-2.18	<0.001
COPD	1.33	0.97-1.81	0.76
Positive CMV serology	1.20	0.91-1.60	0.20
Peripheral vascular disease	2.03	1.39-2.96	<0.001
Mechanical ventilation	0.98	0.74-1.30	0.88

Circulatory support			
No	1		
IABP	0.61	0.42-0.87	0.007
ECMO	1.24	0.84-1.85	0.28
VAD	1.03	0.59-1.80	0.92
Previous sternotomy	1.03	0.81-1.32	0.81
Pretransplant neoplasy	1.37	0.79-2.37	0.26
Post-transplantation TR	1.04	1.01-1.07	p: 0.02
**Donor characteristics**			
Age (years)	1.00	0.99-1.01	0.46
Female sex	1.07	0.88-1.30	0.54
Body mass index (Kg/m^2)	0.99	0.96-1.02	0.53
Positive CMV serology	1.15	0.89-1.48	0.30
Predonation cardiac arrest	0.78	0.50-1.19	0.25
Cause of death			
Trauma	1		
Cerebrovascular accident	1.04	0.83-1.31	0.73
Other	1.00	0.77-1.29	0.99
**Donor-recipient interaction**			
Sex mismatch			
No	1		
Donor male/recipient female	0.89	0.62-1.27	0.52

Donor female/recipient male	1.17	0.93-1.48	0.17
CMV serology mismatch			
No	1		
Donor negative/recipient positive	0.96	0.72-1.26	0.75
Donor positive/recipient negative	1.00	0.74-1.37	0.99
Predicted right ventricular mass ratio	0.99	0.63-1.55	0.95
Predicted total heart mass ratio	0.43	0.21-0.92	0.028
Surgical procedure			
Urgent code	0.80	0.64-1.00	0.049
Cold ischemia duration (min)	1.00	1.00-1.00	0.75
Bicaval surgical technique	0.65	0.49-0.86	0.003

CMV: Cytomegalovirus; COPD: Chronic Obstructive Pulmonary Disease; ECMO: Extracorporeal Membrane Oxygenation; IABP: Intra-Aortic Balloon Pump; TR: Tricuspid regurgitation; VAD: Ventricular Assist Device.
